# Supplementary material for: The tectonigral pathway regulates appetitive locomotion in predatory hunting in mice
Source: Nat Commun. 2021 Jul 20;12:4409. doi: 10.1038/s41467-021-24696-3 (PMC8292483; doi:10.1038/s41467-021-24696-3)
Supplement: Supplementary file 1 — Supplementary Information [file 41467_2021_24696_MOESM1_ESM.pdf]

Supplementary Materials

The tectonigral pathway regulates appetitive locomotion in predatory hunting

Meizhu Huang, Dapeng Li, Xinyu Cheng, Qing Pei, Zhiyong Xie, Huating Gu, Xuerong Zhang, Zijun Chen, Aixue Liu, Yi Wang, Fangmiao Sun, Yulong Li, Jiayi Zhang, Miao He, Yuan Xie, Fan Zhang, Xiangbing Qi, Congping Shang, Peng Cao

This file includes:

Supplementary Fig. 1 to 11

Supplementary Videos 1 to 7

Supplementary Tables 1 to 4

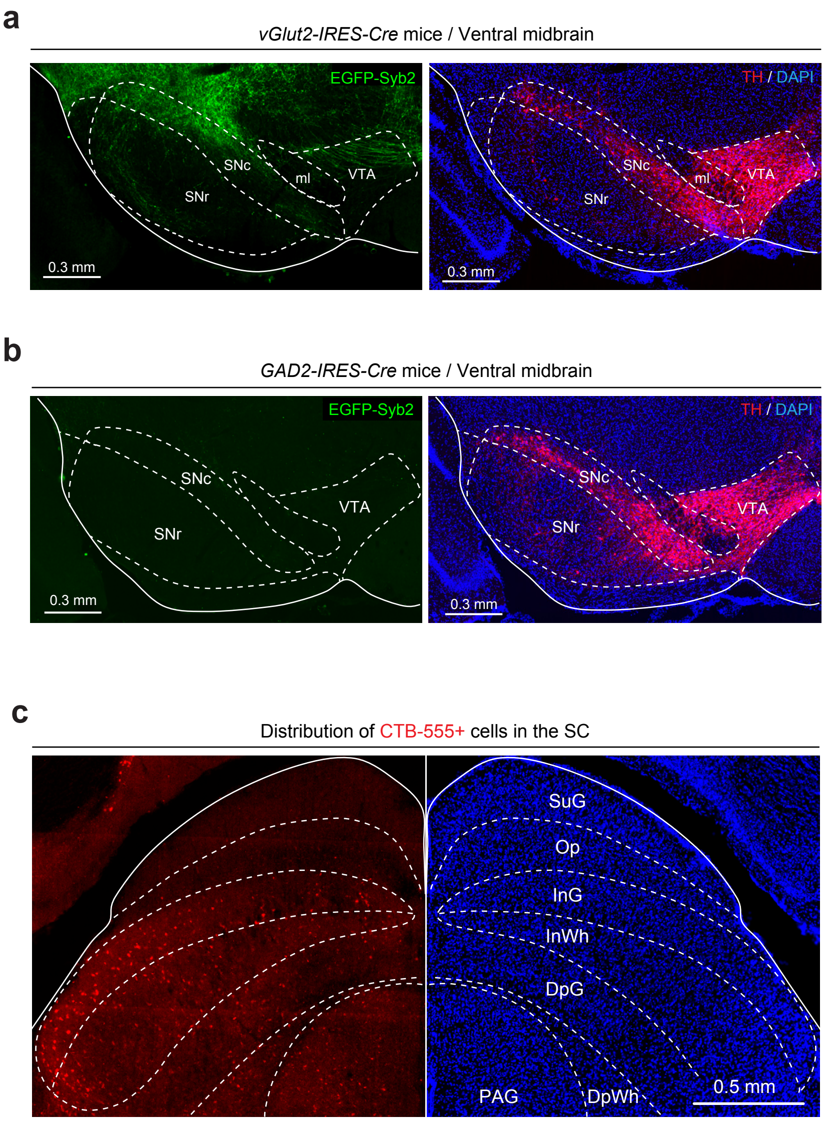

Supplementary Fig. 1 Huang et al., 2021

Supplementary Fig. 1 Cell-type-specific mapping of tectonigral pathway

(a, b) Single-channel and merged micrographs showing EGFP-Syb2+ axon terminals in the ventral midbrain of *vGlut2-IRES-Cre* (a) and *GAD2-IRES-Cre* mice (b). The boundaries of SNc and VTA were determined by immunofluorescence of tyrosine hydroxylase (TH, red). (**c**) An example coronal section of the SC showing the distribution of SNc-projecting SC neurons that were labeled by CTB-555. Scale bars were indicated in the graphs.

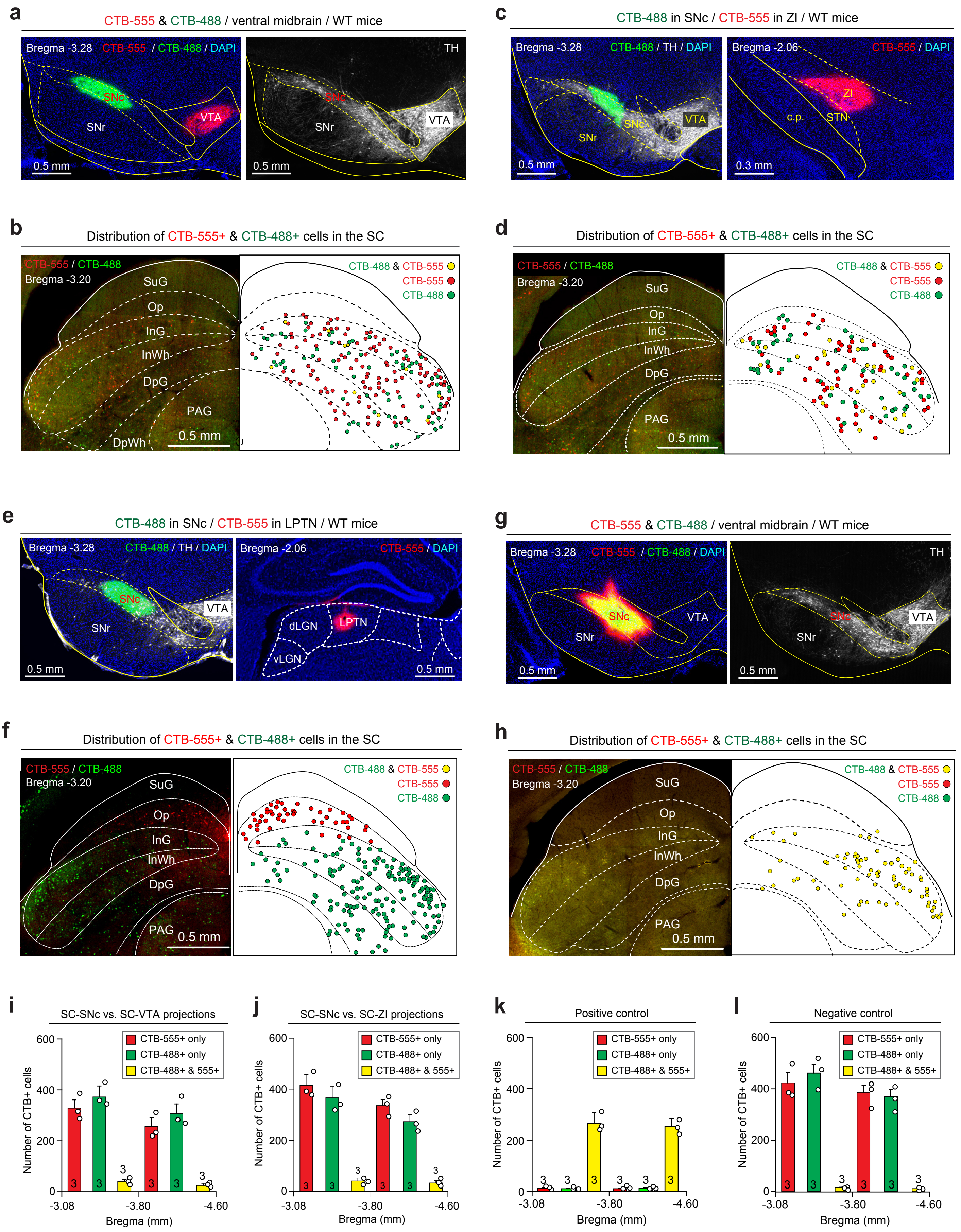

Supplementary Fig. 2 Huang et al., 2021

Supplementary Fig. 2 The SC-SNc pathway is anatomically segregated from other tectofugal pathways

(a) Example coronal section of the ventral midbrain showing injection of CTB-488 and CTB-555 into the SNc and VTA (*left*), the boundaries of which were delineated according to the immunofluorescence of TH (*right*). **(b)** An example coronal section of the SC (*left*) and the corresponding illustration (*right*) showing the distribution of CTB-488+ & CTB-555+ cells in the SC. **(c)** Example coronal brain sections showing injection of CTB-488 and CTB-555 into the SNc (*left*) and ZI (*right*), respectively. **(d)** An example coronal section of the SC (*left*) and the corresponding illustration (*right*) showing the distribution of CTB-488+ & CTB-555+ cells in the SC. **(e)** Example coronal brain sections showing injection of CTB-488 and CTB-555 into the SNc (*left*) and LPTN (*right*), respectively. **(f)** An example coronal section of the SC (*left*) and the corresponding illustration (*right*) showing the distribution of CTB-488+ & CTB-555+ cells in the SC. **(g)** Example coronal section of the ventral midbrain showing injection of mixed CTB-488 & CTB-555 into the SNc (*left*), the boundary of which was delineated according to the immunofluorescence of TH (*right*). (**h**) An example coronal section of the SC (*left*) and the corresponding illustration (*right*) showing the distribution of CTB-488+ & CTB-555+ cells in the SC. **(i-l)** Quantitative analyses of the number of cells labeled by CTB-488 and/or CTB-555 in the anterior SC (from Bregma -3.08 mm to -3.80 mm) and posterior SC (from Bregma -3.80 mm to -4.60 mm), showing how the SC-SNc pathway anatomically relates to the SC-VTA pathway (i), the SC-ZI pathway (j), the SC-LPTN pathway (k), and the SC-SNc pathway itself (as a control, l). Scale bars are labeled in the graphs. Numbers of mice (i-l) are indicated in the graphs. Data in (i-l) are means ± SEM.

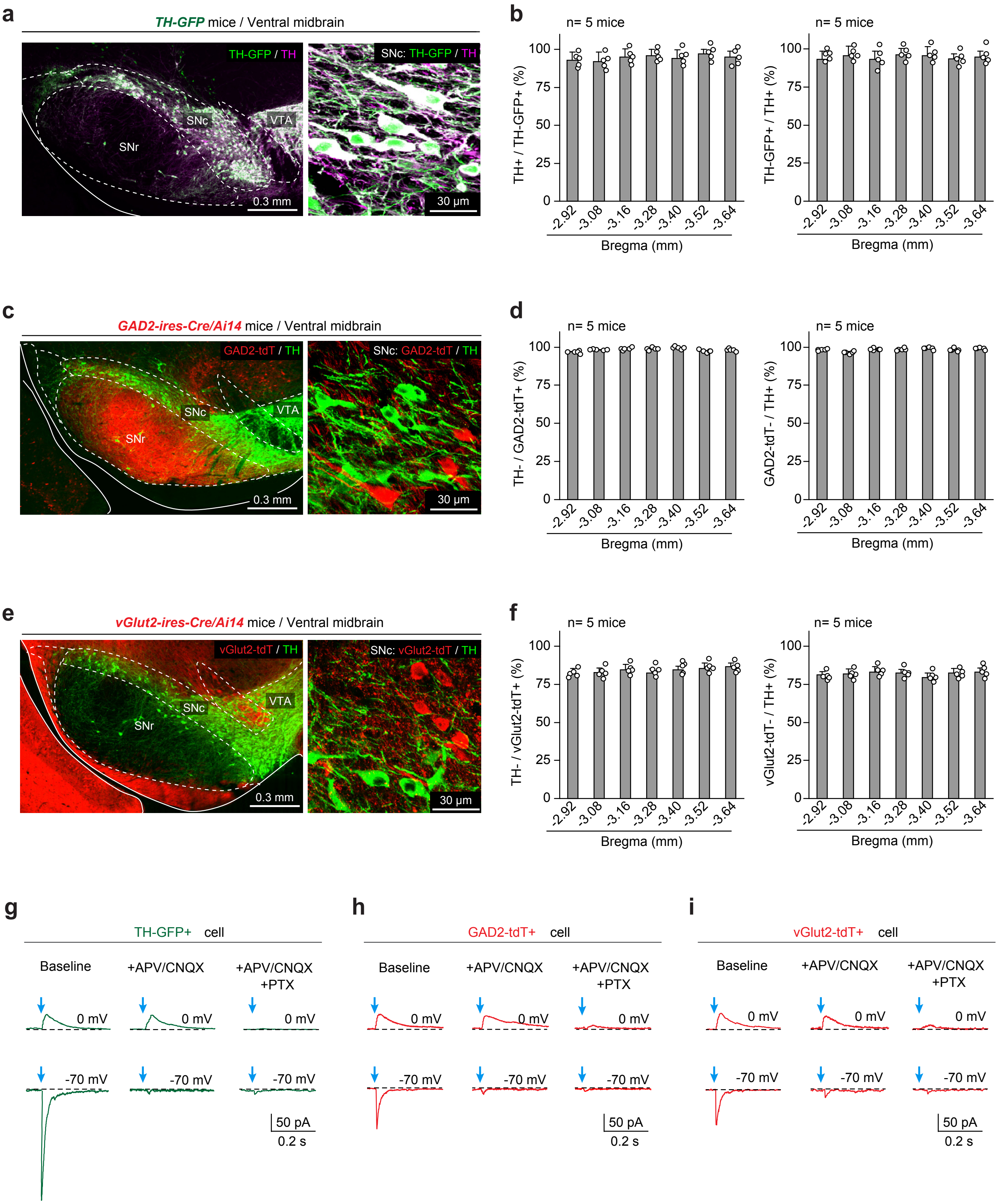

Supplementary Fig. 3 Huang et al., 2021

Supplementary Fig. 3 Dopamine neurons are the primary postsynaptic target of the SC-SNc pathway

(a) Example micrographs showing that TH-GFP+ cells and TH+ cells are largely overlapped in the SNc of *TH-GFP* mice. **(b)** Quantitative analyses showing that the TH-GFP+ cells in the SNc at different bregma were predominantly TH+ (*left*), whereas most TH+ cells were TH-GFP+ (*right*). **(c)** Example micrographs showing that GAD2-tdT+ cells and TH+ cells are largely segregated in the SNc of *GAD2-IRES-Cre/Ai14* mice. **(d)** Quantitative analyses showing that the GAD2-tdT+ cells in the SNc at different bregma were predominantly TH- (*left*), whereas most TH+ cells were GAD2-tdT- (*right*). **(e)** Example micrographs showing that vGlut2-tdT+ cells and TH+ cells are largely segregated in the SNc of *vGlut2-IRES-Cre/Ai14* mice. **(f)** Quantitative analyses showing that the most vGlut2-tdT+ cells in the SNc at different bregma were TH- (*left*), whereas TH+ cells were predominantly vGlut2-tdT- (*right*). (**g-i**) Example traces of oIPSCs (*top*) and oEPSCs (*bottom*) from putative SNc dopamine neurons (TH-GFP+) (g), putative GAD2+ neurons (GAD2-tdT+) (h) and putative vGlut2+ neurons (vGlut2-tdT+) (i) with and without perfusion of antagonists of glutamate receptors (APV/CNQX) and GABAa receptor (PTX). Numbers of mice (b, d, f) are indicated in the graphs. Data in (b, d, f) are means ± SEM. Scale bars are labeled in the graphs.

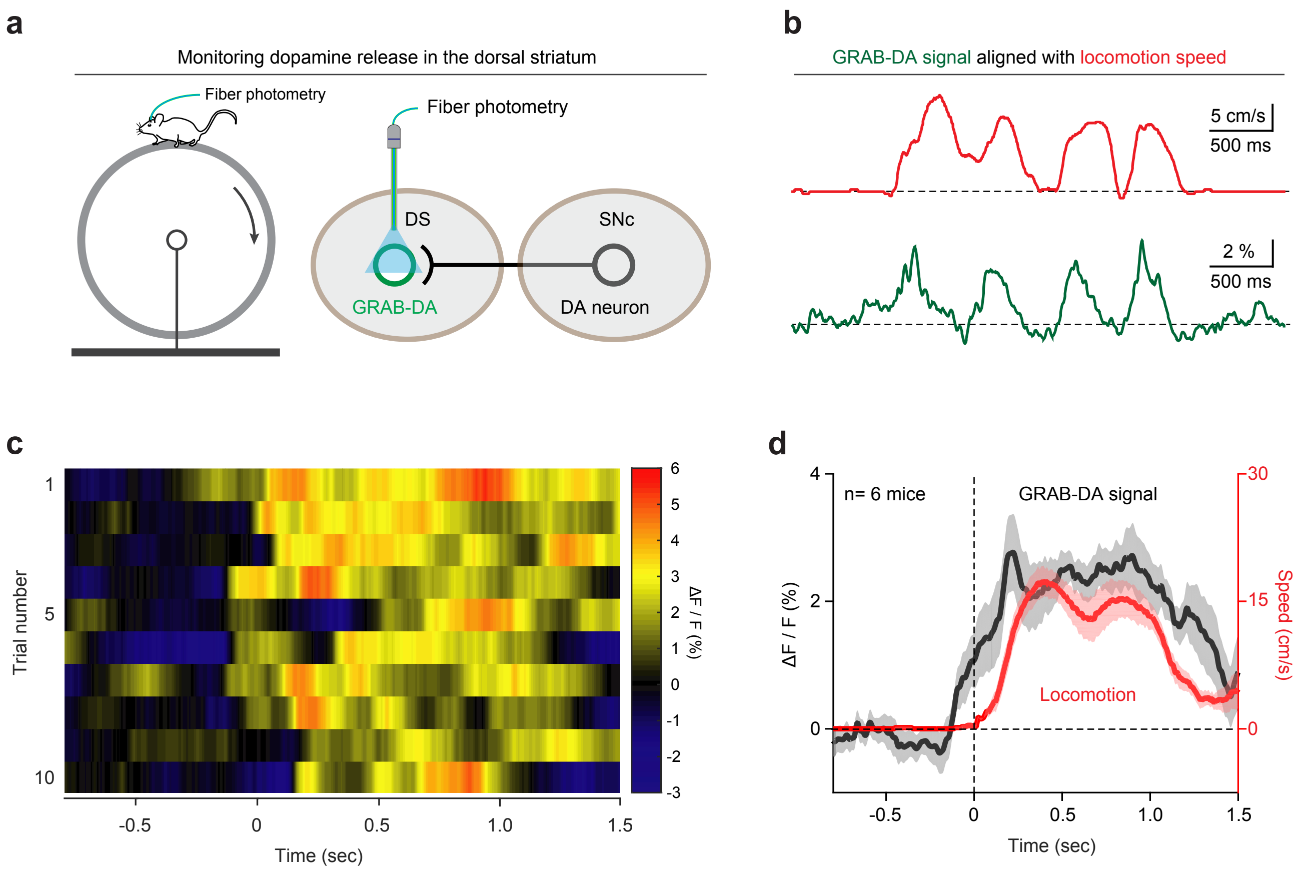

Supplementary Fig. 4 Huang et al., 2021

Supplementary Fig. 4 $GRAB_{DA}$ signals from the dorsal striatum

(a) Schematic diagram showing fiber photometry recording of $GRAB_{DA}$ signals from dorsal-striatal neurons expressing $GRAB_{DA}$ sensor in a head-fixed mouse walking on a cylindrical treadmill. **(b)** Example traces of $GRAB_{DA}$ signal (*bottom*) aligned with locomotion speed (*top*). **(c, d)** Heat-map graphs of individual $GRAB_{DA}$ signals (c) and averaged $GRAB_{DA}$ response curve (d) aligned with locomotion initiation (Time = 0 sec), showing the temporal relationship between $GRAB_{DA}$ signals and locomotion initiation. Numbers of mice are indicated in the graphs (d). Data in (d) are means ± SEM (error bars). Scale bars are labeled in the graphs.

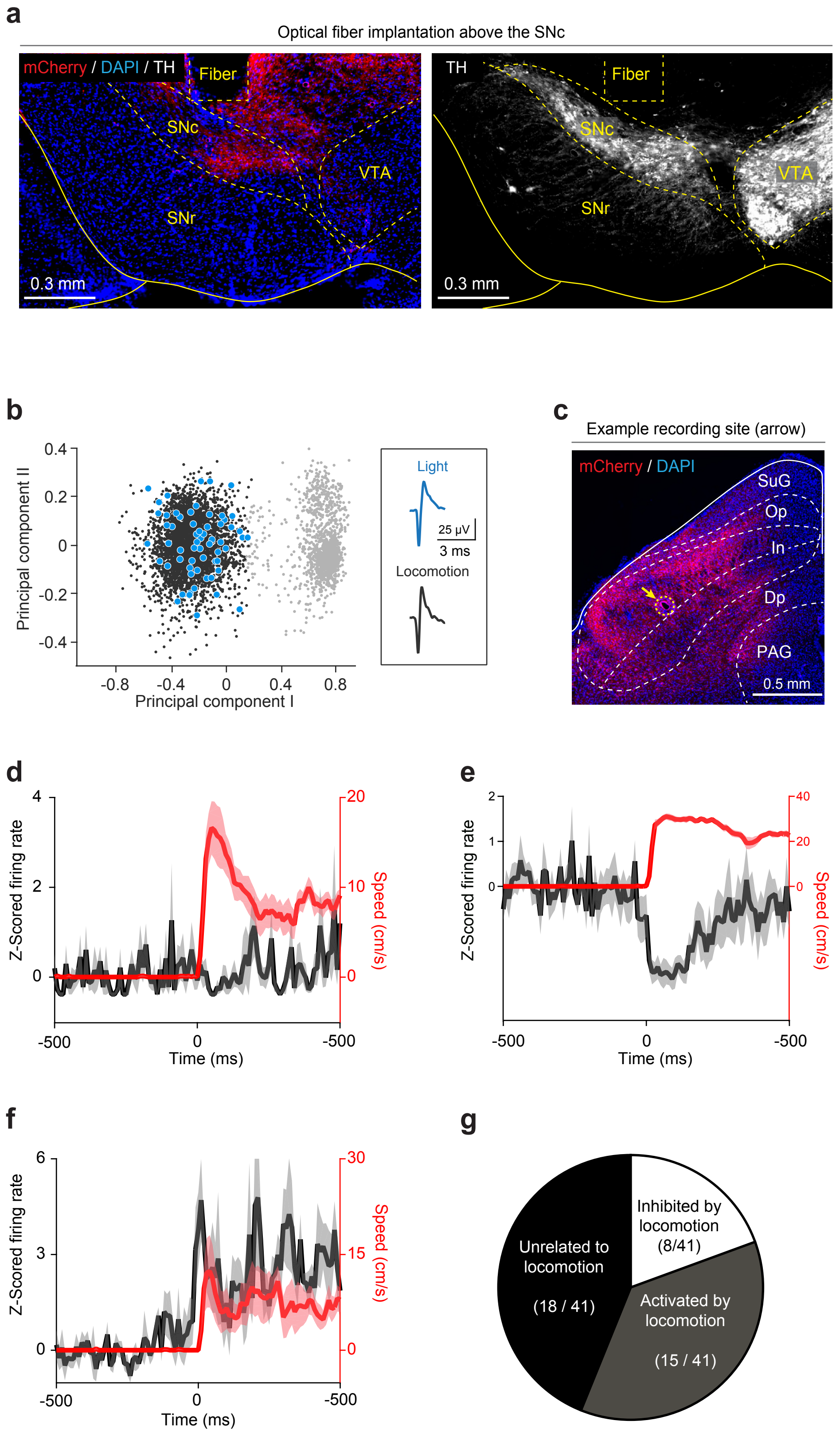

Supplementary Fig. 5 Huang et al., 2021

Supplementary Fig. 5 SNc-projecting SC neurons encode the speed of locomotion

(**a**) Example coronal section of the ventral midbrain showing the optical-fiber track above ChR2-mCherry^{+} axon terminals in the SNc (*left*), the boundary of which was determined by immunofluorescence of TH (*right*). **(b)** Principal component analyses of light-evoked spikes (blue) and locomotion-evoked spikes (black) of an example putative SNc-projecting SC neuron. Gray dots, noise. **(c)** Example coronal section of the SC showing a recording site marked by electrolytic lesion (arrow) in the intermediate layer (In) of the SC. **(d-f)** Three example units that were not identified as SNc-projecting SC neurons, showing unchanged (d), decreased (e) and increased (f) firing rate (black) when locomotion was initiated (red). **(g)** Summary of the 41 units that were not identified as SNc-projecting SC neurons, showing various types of relationship between firing rate and locomotion initiation. Data in (d-f) are means ± SEM (error bars). Scale bars are labeled in the graphs.

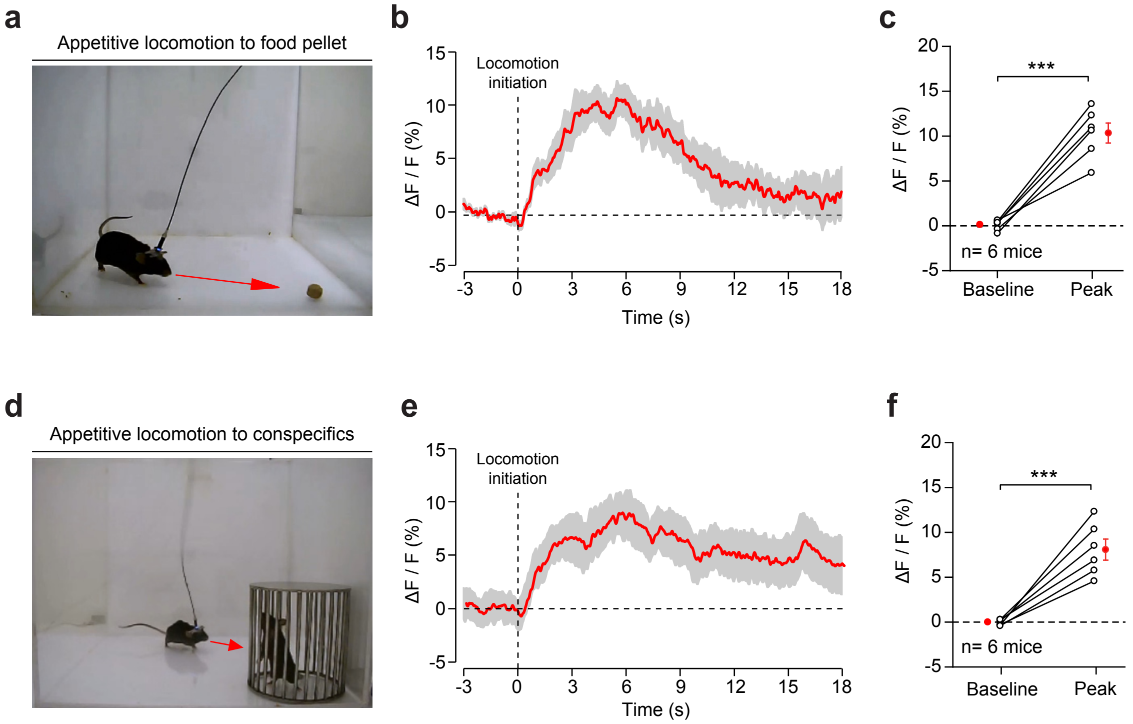

Supplementary Fig. 6 Huang et al., 2021

Supplementary Fig. 6 Fiber photometry recording from the SNc-projecting SC neurons

(a, b) Example picture (a) and time course of normalized GCaMP fluorescence (b) of an example mouse before and during its appetitive locomotion toward food pellet. **(c)** Quantitative analyses of normalized GCaMP fluorescence showing the activity of SNc-projecting SC neurons was significantly increased during appetitive locomotion toward food pellet. **(d, e)** Example picture (d) and time course of normalized GCaMP fluorescence (e) of an example mouse before and during its appetitive locomotion toward conspecifics. **(f)** Quantitative analyses of normalized GCaMP fluorescence showing the activity of SNc-projecting SC neurons was significantly increased during appetitive locomotion toward conspecifics. Numbers of mice are indicated in the graphs (c, f). Data in (b, c, e, f) are means ± SEM (error bars). For the P values, see Supplementary Table 4.

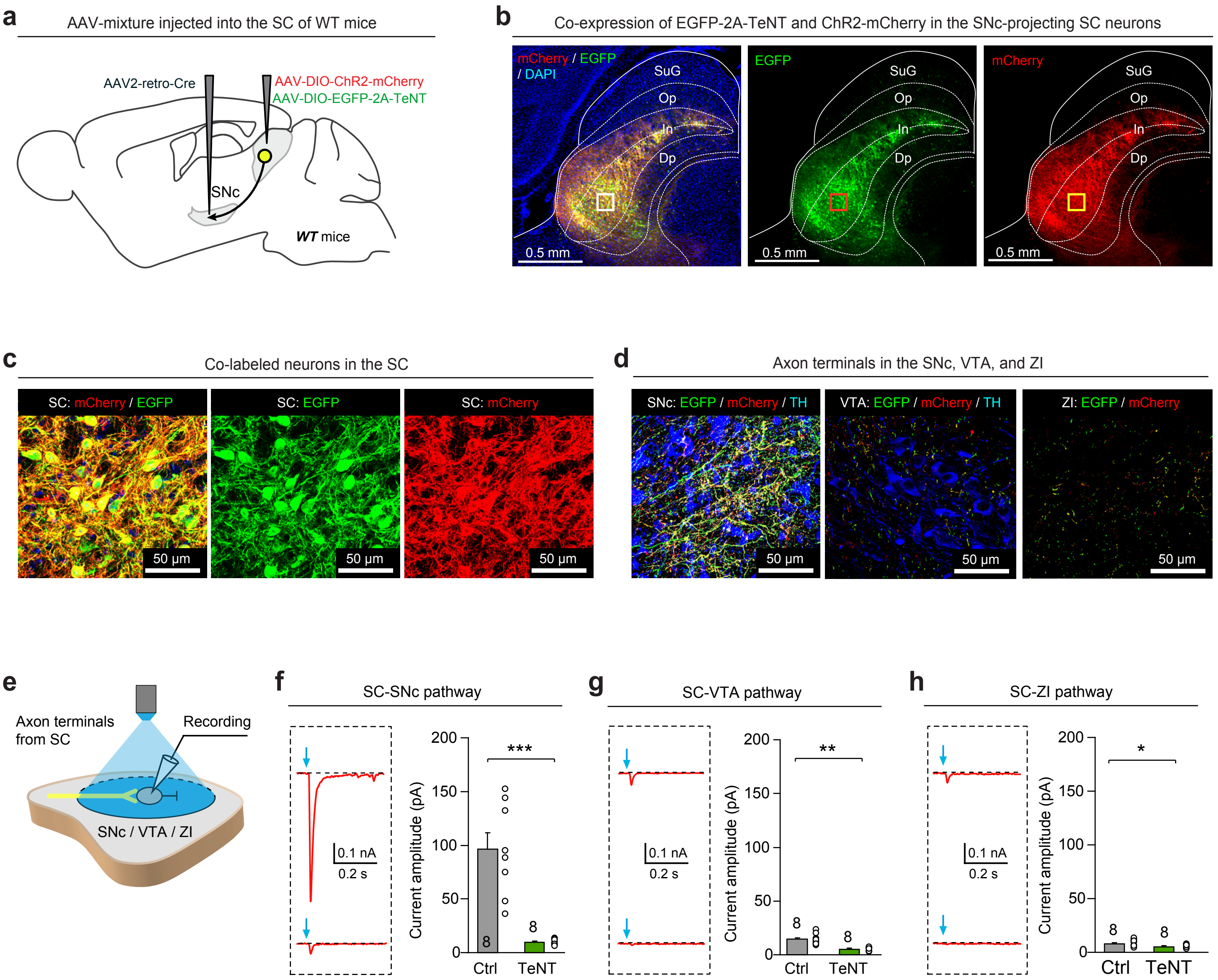

Supplementary Fig. 7 Huang et al., 2021

Supplementary Fig. 7 Control experiment to verify TeNT-mediated synaptic inactivation of the SNc-projecting SC neurons with the dual AAV strategy

(a) Schematic diagram showing the dual-AAV strategy to express ChR2-mCherry and TeNT in the SNc-projecting SC neurons of WT mice. **(b, c)** An example coronal brain section of the SC (b) and a micrograph from the SC (c) showing co-expression of ChR2-mCherry and TeNT in the same SNc-projecting SC neurons. **(d)** Example micrographs from the SNc (*left*), VTA (*middle*), and ZI (*right*), showing axons of SNc-projecting SC neurons in these brain areas. **(e)** Schematic diagram showing whole-cell recording of light-evoked postsynaptic currents from the neurons in the SNc, VTA or ZI in acute brain slices. **(f-h)** Example traces (*left*) and quantitative analyses (*right*) showing the effects of TeNT on the amplitude of light-evoked PSCs. Numbers of cells (f-h) are indicated in the graphs. Data in (f-h) are means ± SEM. Statistic analyses (f-h) were performed using Student t-test (*** $P<0.001$; **$P<0.01$; * $P<0.05$). For the P values, see Supplementary Table 4. Scale bars are labeled in the graphs.

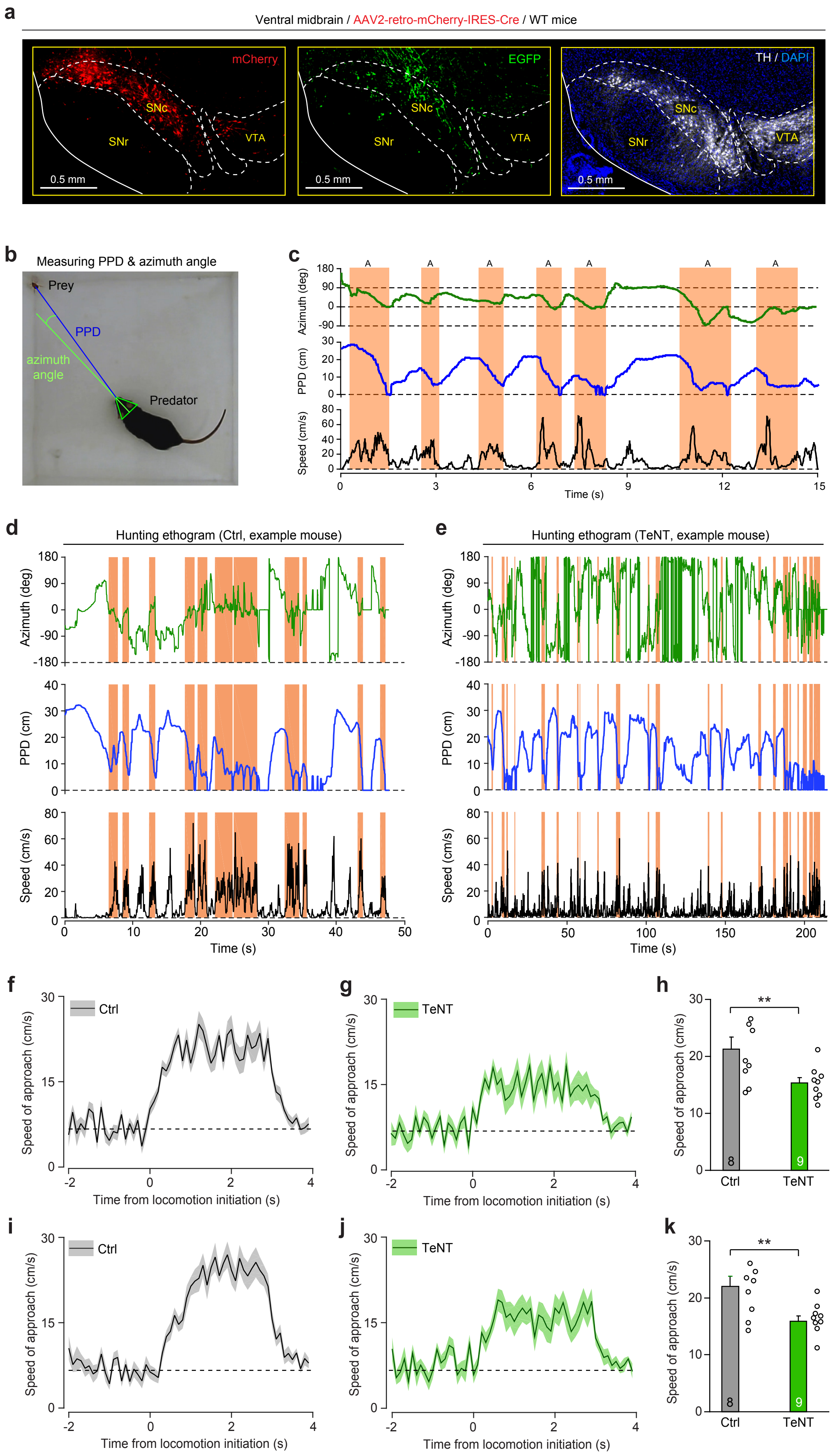

Supplementary Fig. 8 Huang et al., 2021

Supplementary Fig. 8 Effects of TeNT-mediated synaptic inactivation of the SC-SNc pathway on appetitive locomotion

(a) Example coronal section of ventral midbrain showing mCherry+ cells in the SNc (*left*) were intermingled with EGFP+ axon terminals from SC neurons (*middle*). The boundary of the SNc was determined by immunofluorescence of TH (*right*). **(b)** An example picture showing computer-aided measurement of azimuth angle and prey-predator distance (PPD) when predator approached prey. **(c)** Aligned time courses of azimuth angle (*top*), prey-predator distance (PPD, *middle*), and locomotion speed (*bottom*) of an example mouse in predatory hunting, showing the identification of approach episodes (shaded areas in orange). The intermittent approach episodes were characterized by azimuth within a narrow range (-90 – 90 deg), by decreased PPD and by pulses of locomotion speed. For the detailed criteria to identify the approach episodes, see Methods. **(d, e)** Time courses of azimuth angle (*top*), PPD (*middle*), and locomotion speed (*bottom*) during predatory hunting of example mice without (d, Ctrl) and with (e, TeNT) synaptic inactivation of the SNc-projecting SC neurons. **(f, g)** Time courses of locomotion speed of an example mouse before and during appetitive locomotion toward food pellet without (Ctrl, f) and with (TeNT, g) synaptic inactivation of SNc-projecting SC neurons. **(h)** Quantitative analyses of average locomotion speed during appetitive locomotion toward food pellet in mice without (Ctrl) and with (TeNT) synaptic inactivation of SNc-projecting SC neurons. **(i, j)** Time courses of locomotion speed of an example mouse before and during appetitive locomotion toward conspecifics without (Ctrl, i) and with (TeNT, j) synaptic inactivation of SNc-projecting SC neurons. **(k)** Quantitative analyses of average locomotion speed during appetitive locomotion toward conspecifics in mice without (Ctrl) and with (TeNT) synaptic inactivation of SNc-projecting SC neurons. Scale bars are labeled in the graphs (a). Numbers of mice are indicated in the graphs (h, k). Data in (f-k) are means ± SEM. Statistic analyses (h, k) are performed using Student t-test (** $P<0.01$). For the P values, see Supplementary Table 4.

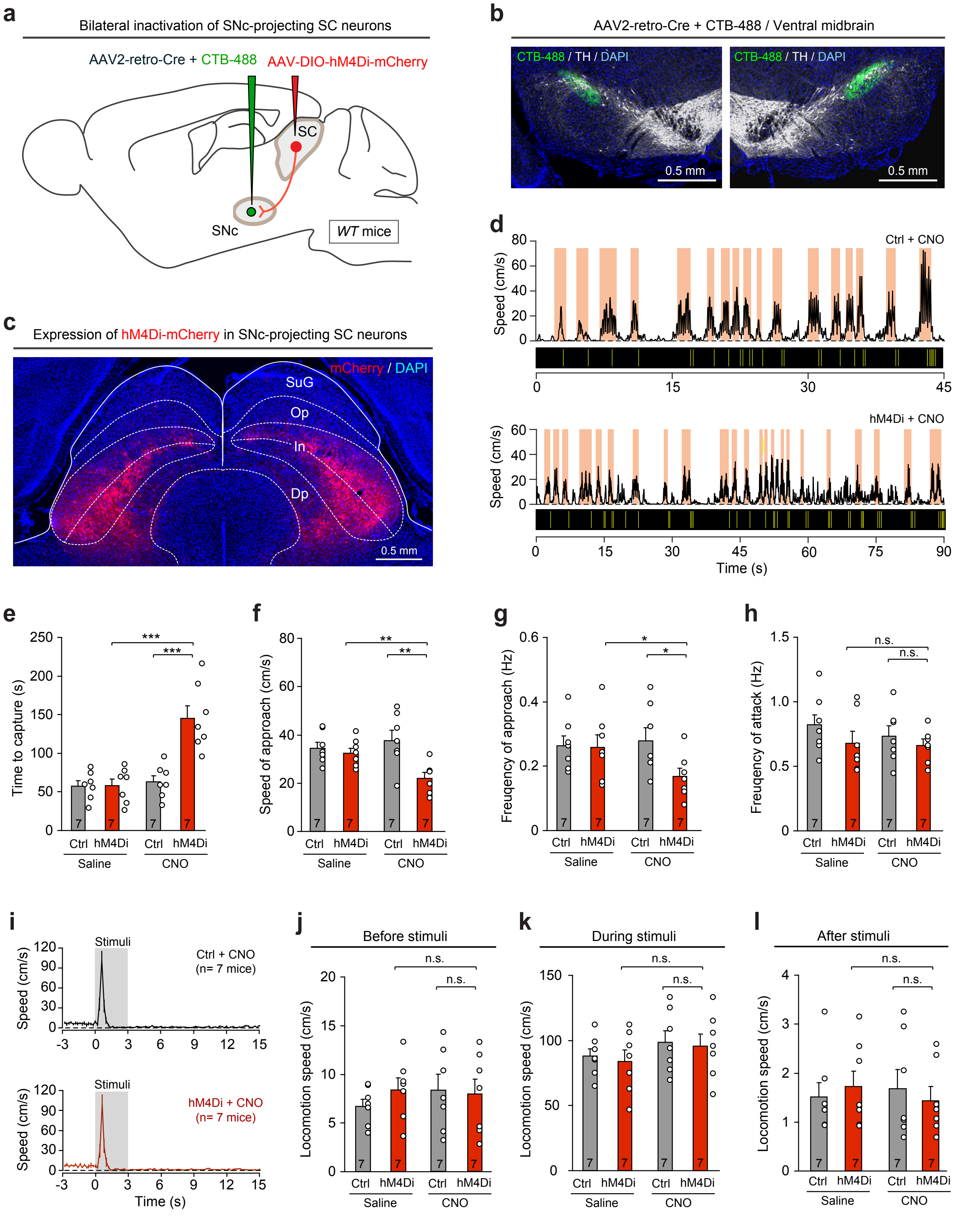

Supplementary Fig. 9 Huang et al., 2021

Supplementary Fig. 9 Effects of chemogenetic inactivation of the SC-SNc pathway on appetitive and defensive locomotion

(a) Schematic diagram showing the dual-AAV strategy to express hM4Di-mCherry in SNc-projecting SC neurons. **(b)** Example coronal brain sections showing the bilateral injection sites of AAV2-retro-Cre mixed with CTB-488. **(c)** Example coronal brain section showing the distribution of hM4Di-mCherry+ SNc-projecting neurons in the SC. **(d)** Behavioral ethograms of predatory hunting of example mice without (Ctrl+CNO) and with (hM4Di+CNO) chemogenetic inactivation of the SC-SNc pathway. **(e-h)** Quantitative analyses of hunting behavior showing the effects of chemogenetic inactivation of the SNc-projecting SC neurons on time to capture (e), speed of approach (f), frequency of approach (g), and frequency of predatory attack (h). **(i)** Time courses of locomotion speed before, during and after looming visual stimuli in mice without (Ctrl+CNO) and with (hM4Di+CNO) chemogenetic inactivation of the SC-SNc pathway. **(j-l)** Quantitative analyses of locomotion speed before (j), during (k) and after (l) looming visual stimuli, showing chemogenetic inactivation of the SC-SNc pathway had little effect on defensive locomotion. Scale bars are labeled in the graphs (b, c). Numbers of mice are indicated in the graphs (e-l). Data in (e-l) are means ± SEM. Statistic analyses (e-h, j-l) are performed using Student t-test (** $P<0.01$). For the P values, see Supplementary Table 4.

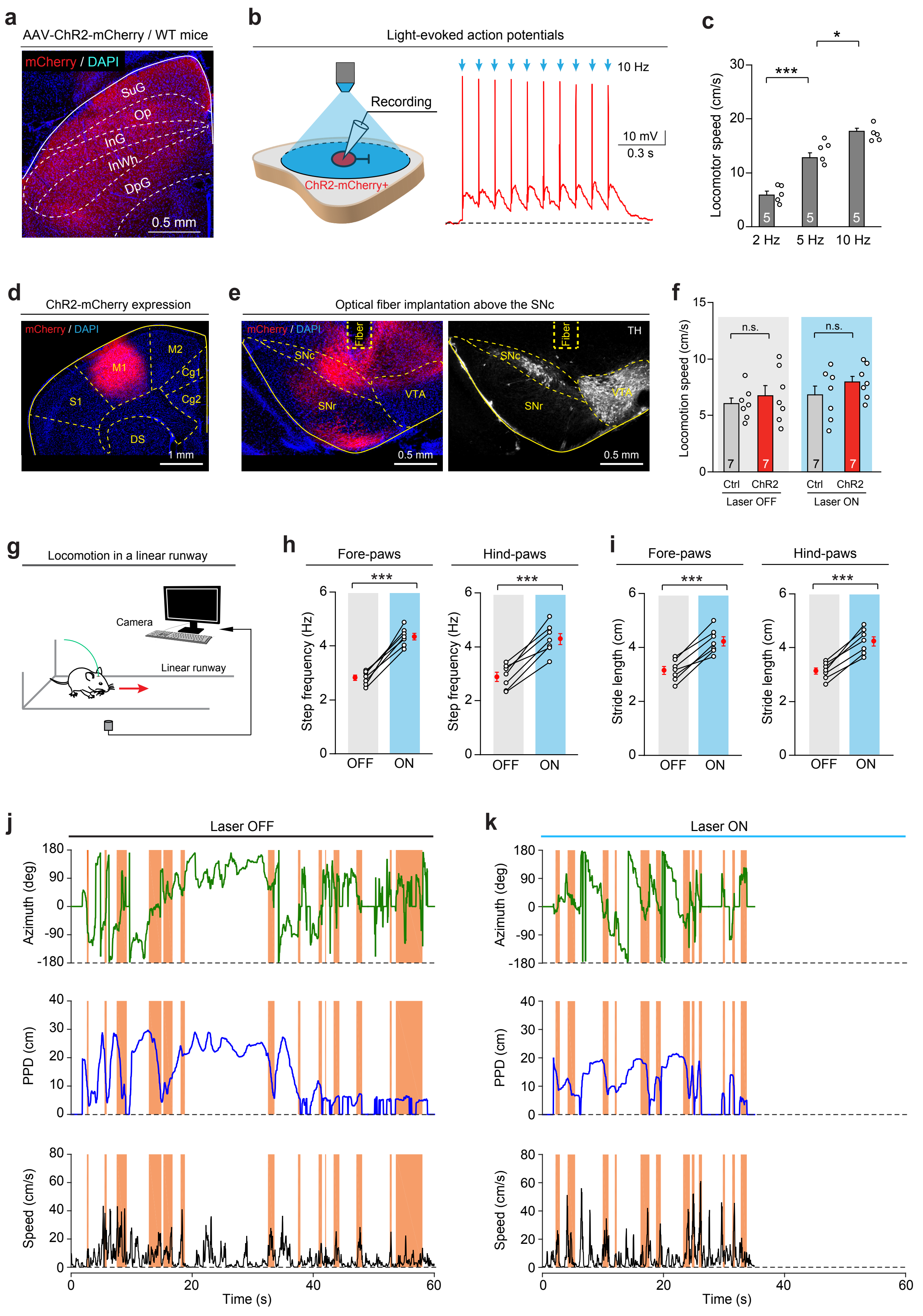

Supplementary Fig. 10 Huang et al., 2021

Supplementary Fig. 10 Activation of the SC-SNc pathway promotes appetitive locomotion during predatory hunting

(**a**) Example coronal section of the SC showing ChR2-mCherry expression in the SC of WT mice. (**b**) Schematic diagram (*left*) and example trace (*right*) showing light pulses (2 ms, 473 nm, 10 Hz, 10 pulses) reliably triggered action potential firing from ChR2-mCherry$^+$ SC neurons in acute SC slices. **(c)** Quantitative analyses of average locomotion speed of mice in the linear runway during photostimulation of the SC-SNc pathway (10 ms, 6 s, 10 mW) with different frequencies (2 Hz, 5 Hz, 10 Hz). **(d, e)** AAV-ChR2-mCherry was injected into the primary motor cortex (M1) of *WT* mice (d), followed by an optical fiber implanted above the ChR2-mCherry+ axon terminals in the SNc (e). **(f)** Quantitative analyses of locomotion speed on the linear runway without (OFF) and with (ON) light stimulation of M1-SNc pathway (10 Hz, 10 ms, 10 mW). **(g)** Schematic diagram showing the experimental configuration to monitor paw movements of mice walking on the linear runway. (**h, i**) Quantitative analyses of step frequency (h) and stride length (i) of fore-paws (*left*) or hind-paws (*right*) in mice walking on the linear runway without (OFF) and with (ON) activation of the SC-SNc pathway (10 Hz, 10 ms, 10 mW). (**j, k**) Time courses of azimuth angle (*top*), PPD (*middle*), and locomotion speed (*bottom*) during predatory hunting of an example mouse without (j, Laser OFF) and with (k, Laser ON) photostimulation of the SC-SNc pathway. Numbers of mice (c, f, h, i) are indicated in the graphs. Data in (c, f, h, i) are means ± SEM. Statistic analyses (c, f, h, i) were performed using Student t-test (*** $P<0.001$; * $P<0.05$; n.s. $P>0.1$). For the P values, see Supplementary Table 4. Scale bars are labeled in the graphs.

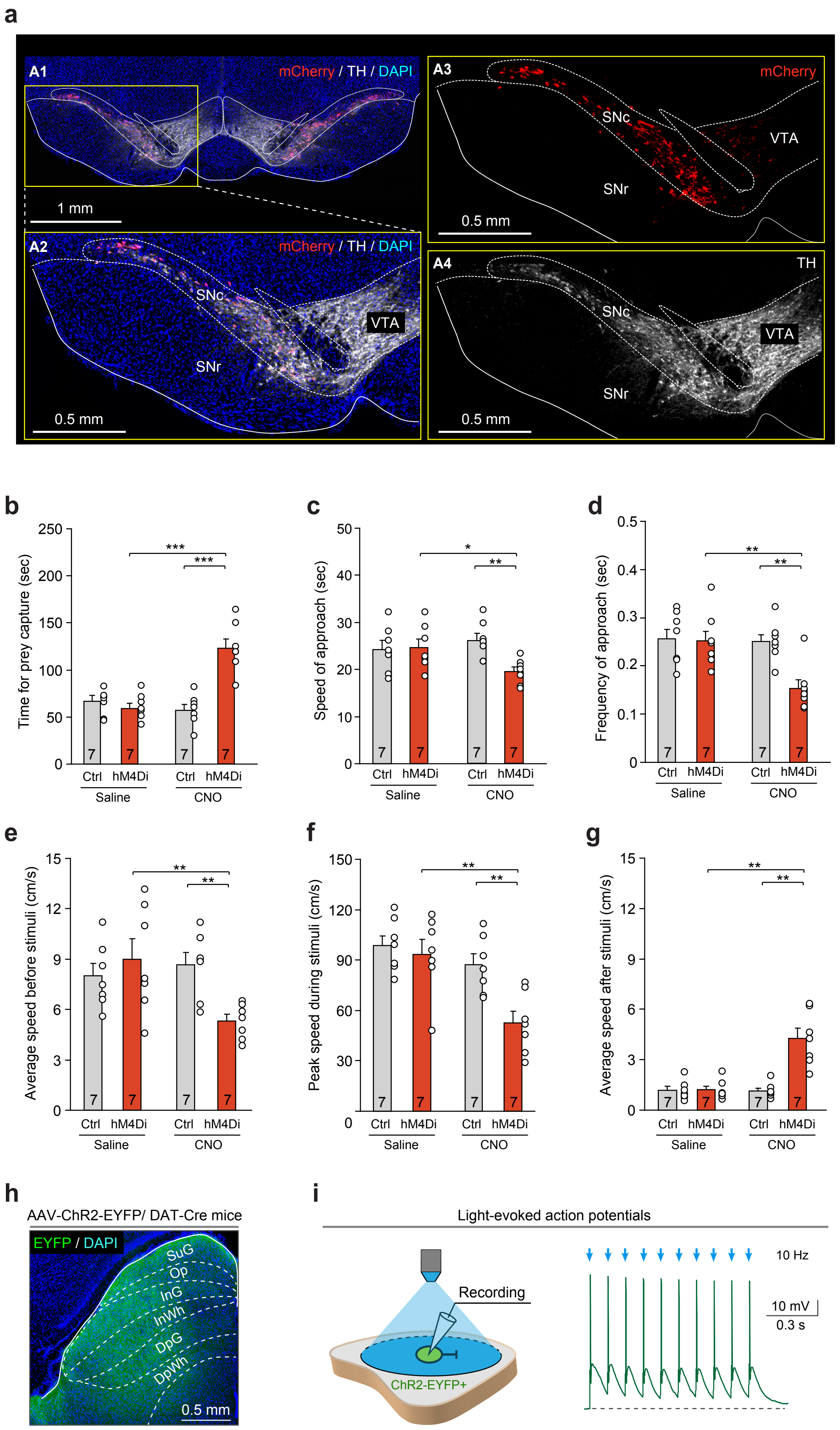

Supplementary Fig. 11 Huang et al., 2021

Supplementary Fig. 11 Effects of chemogenetic inactivation of SNC dopamine neurons on basal predatory hunting in mice

(**a**) Example coronal brain section showing injection of AAV-DIO-hM4Di-mCherry in the bilateral SNc of DAT-Cre mice (a1), which resulted in localized expression of hM4Di-mCherry in the SNc with little leak to the adjacent VTA (A2-A4). (**b-d**) Quantitative analyses of chemogenetic inactivation of SNc dopamine neurons on the time for prey capture (b), speed of approach (c), and frequency of approach (d) during predatory hunting in mice. (**e-g**) The effects of chemogenetic inactivation of SNc dopamine neurons on the locomotion speed before, during and after looming visual stimuli. (**h**) Example coronal section showing ChR2-EYFP expression in the SC. (**I**) Schematic diagram (*left*) and example trace (*right*) showing light pulses (2 ms, 473 nm, 10 Hz, 10 pulses) reliably triggered action potential firing from ChR2-EYFP+ SC neurons in acute SC slices. Numbers of mice (b-g) are indicated in the graphs. Data in (b-g) are means ± SEM. Statistic analyses (b-g) were performed using Student t-test (** P<0.01). For the P values, see Supplementary Table 4. Scale bars are labeled in the graphs.

Supplementary Video 1 Action potential firing of SNc-projecting SC neurons during locomotion

This movie shows that the action potential firing of a putative SNc-projecting SC neuron recorded with an optrode is correlated with locomotion. The action potentials have been sorted and their waveforms are displayed in the corner of the movie.

Supplementary Video 2 Predatory hunting of an example control mouse without synaptic inactivation of SNc-projecting SC neurons

This movie shows behavioral analyses of predatory hunting of an example control mouse without synaptic inactivation of SNc-projecting SC neurons. The left part of the screen displays the video taken by the overhead camera in parallel with computer-aided analyses of azimuth angle and PPD in real-time. The right part of the screen displays the time courses of azimuth angle, locomotion speed and PPD during predatory hunting in real-time. The approach episodes were labeled with shaded areas in orange.

Supplementary Video 3 Predatory hunting of an example test mouse with synaptic inactivation of SNc-projecting SC neurons

This movie shows behavioral analyses of predatory hunting of an example test mouse with synaptic inactivation of SNc-projecting SC neurons. The left part of the screen displays the video taken by the overhead camera in parallel with computer-aided analyses of azimuth angle and PPD in real-time. The right part of the screen displays the time courses of azimuth angle, locomotion speed and PPD during predatory hunting in real-time. The approach episodes were labeled with shaded areas in orange.

Supplementary Video 4 Defensive locomotion of an example control mouse without synaptic inactivation of SNc-projecting SC neurons

This movie shows the overhead looming visual stimuli triggered escape followed by long-lasting freezing in an example control mouse without synaptic inactivation of SNc-projecting SC neurons.

Supplementary Video 5 Defensive locomotion of an example test mouse with synaptic inactivation of SNc-projecting SC neurons

This movie shows the overhead looming visual stimuli evoked immediate escape followed by long-lasting freezing in an example test mouse with synaptic inactivation of SNc-projecting SC neurons.

Supplementary Video 6 Predatory hunting of an example control mouse without photostimulation of the toctoginral pathway pathway

This movie shows behavioral analyses of predatory hunting of an example control mouse without photostimulation of the SC-SNc pathway. The left part of the screen displays the video taken by the overhead camera in parallel with computer-aided analyses of azimuth angle and PPD in real-time. The right part of the screen displays the time courses of azimuth angle, locomotion speed and PPD during predatory hunting in real-time. The approach episodes were labeled with shaded areas in orange.

Supplementary Video 7 Predatory hunting of an example test mouse with photostimulation of the SC-SNc pathway

This movie shows behavioral analyses of predatory hunting of an example test mouse with photostimulation of the SC-SNc pathway. The left part of the screen displays the video taken by the overhead camera in parallel with computer-aided analyses of azimuth angle and PPD in real-time. The right part of the screen displays the time courses of azimuth angle, PPD and locomotion speed during predatory hunting in real-time. The approach episodes were labeled with shaded areas in orange.

Supplementary Table 1 Mouse lines and reagents

Supplementary Table 2 Summary of all experimental designs

Supplementary Table 3 Summary of cell counting strategy

Supplementary Table 4 Summary of statistical analyses
