# Supplementary material for: The tectonigral pathway regulates appetitive locomotion in predatory hunting in mice
Source: Nat Commun. 2021 Jul 20;12:4409. doi: 10.1038/s41467-021-24696-3 (PMC8292483; doi:10.1038/s41467-021-24696-3)
Supplement: Supplementary file 3 — Description of Additional Supplementary Files [file 41467_2021_24696_MOESM3_ESM.pdf]

Supplementary Video 1 Action potential firing of SNc-projecting SC neurons during locomotion

This movie shows that the action potential firing of a putative SNc-projecting SC neuron recorded with an optrode is correlated with locomotion. The action potentials have been sorted and their waveforms are displayed in the corner of the movie.

Supplementary Video 2 Predatory hunting of an example control mouse without synaptic inactivation of SNc-projecting SC neurons

This movie shows behavioral analyses of predatory hunting of an example control mouse without synaptic inactivation of SNc-projecting SC neurons. The left part of the screen displays the video taken by the overhead camera in parallel with computer-aided analyses of azimuth angle and PPD in real-time. The right part of the screen displays the time courses of azimuth angle, locomotion speed and PPD during predatory hunting in real-time. The approach episodes were labeled with shaded areas in orange.

Supplementary Video 3 Predatory hunting of an example test mouse with synaptic inactivation of SNc-projecting SC neurons

This movie shows behavioral analyses of predatory hunting of an example test mouse with synaptic inactivation of SNc-projecting SC neurons. The left part of the screen displays the video taken by the overhead camera in parallel with computer-aided analyses of azimuth angle and PPD in real-time. The right part of the screen displays the time courses of azimuth angle, locomotion speed and PPD during predatory hunting in real-time. The approach episodes were labeled with shaded areas in orange.

Supplementary Video 4 Defensive locomotion of an example control mouse without synaptic inactivation of SNc-projecting SC neurons

This movie shows the overhead looming visual stimuli triggered escape followed by long-lasting freezing in an example control mouse without synaptic inactivation of SNc-projecting SC neurons.

Supplementary Video 5 Defensive locomotion of an example test mouse with synaptic inactivation of SNc-projecting SC neurons This movie shows the overhead looming visual stimuli evoked immediate escape followed by long-lasting freezing in an example test mouse with synaptic inactivation of SNc-projecting SC neurons.

Supplementary Video 6 Predatory hunting of an example control mouse without photostimulation of the SC-SNc pathway

This movie shows behavioral analyses of predatory hunting of an example control mouse without photostimulation of the SC-SNc pathway. The left part of the screen displays the video taken by the overhead camera in parallel with computer-aided analyses of azimuth angle and PPD in real-time. The right part of the screen displays the time courses of azimuth angle, locomotion speed and PPD during predatory hunting in real-time. The approach episodes were labeled with shaded areas in orange.

Supplementary Video 7 Predatory hunting of an example test mouse with photostimulation of the SC-SNc pathway

This movie shows behavioral analyses of predatory hunting of an example test mouse with photostimulation of the SC-SNc pathway. The left part of the screen displays the video taken by the overhead camera in parallel with computer-aided analyses of azimuth angle and PPD in real-time. The right part of the screen displays the time courses of azimuth angle, PPD and locomotion speed during predatory hunting in real-time. The approach episodes were labeled with shaded areas in orange.
