# Supplementary material for: The tectonigral pathway regulates appetitive locomotion in predatory hunting in mice
Source: Nat Commun. 2021 Jul 20;12:4409. doi: 10.1038/s41467-021-24696-3 (PMC8292483; doi:10.1038/s41467-021-24696-3)
Supplement: Supplementary file 11 — Reporting Summary [file 41467_2021_24696_MOESM11_ESM.pdf]

nature research

Corresponding author(s): Peng Cao

Last updated by author(s): 6/14/2021

Reporting Summary

Nature Research wishes to improve the reproducibility of the work that we publish. This form provides structure for consistency and transparency in reporting. For further information on Nature Research policies, see our Editorial Policies and the Editorial Policy Checklist.

Statistics

For all statistical analyses, confirm that the following items are present in the figure legend, table legend, main text, or Methods section.

n/a	Confirmed	
☐	☒	The exact sample size (n) for each experimental group/condition, given as a discrete number and unit of measurement
☐	☒	A statement on whether measurements were taken from distinct samples or whether the same sample was measured repeatedly
☐	☒	The statistical test(s) used AND whether they are one- or two-sided<br>*Only common tests should be described solely by name; describe more complex techniques in the Methods section.*
☒	☐	A description of all covariates tested
☐	☒	A description of any assumptions or corrections, such as tests of normality and adjustment for multiple comparisons
☐	☒	A full description of the statistical parameters including central tendency (e.g. means) or other basic estimates (e.g. regression coefficient) AND variation (e.g. standard deviation) or associated estimates of uncertainty (e.g. confidence intervals)
☐	☒	For null hypothesis testing, the test statistic (e.g. F, t, r) with confidence intervals, effect sizes, degrees of freedom and P value noted<br>*Give P values as exact values whenever suitable.*
☒	☐	For Bayesian analysis, information on the choice of priors and Markov chain Monte Carlo settings
☒	☐	For hierarchical and complex designs, identification of the appropriate level for tests and full reporting of outcomes
☒	☐	Estimates of effect sizes (e.g. Cohen's d, Pearson's r), indicating how they were calculated

Our web collection on statistics for biologists contains articles on many of the points above.

Software and code

Policy information about availability of computer code

Data collection	Fiber photometry: commercialized software from ThinkerTech. Single-unit data: commercialized Spike2 Software Version 7.03. Whole-cell recording data: Commercialized Software Clampex 10.5.
Data analysis	Origin Professional 6.0<br>MatLab R2014A<br>ImageJ (FIJI) (version 2.0.0)<br>Clampfit 10.5<br>EthoVision XT 14<br>Spike2 Software Version 7.03<br>Custom code to analyze defensive behavior. Custom code is available upon request.

For manuscripts utilizing custom algorithms or software that are central to the research but not yet described in published literature, software must be made available to editors and reviewers. We strongly encourage code deposition in a community repository (e.g. GitHub). See the Nature Research guidelines for submitting code & software for further information.

April 2020

Data

Policy information about availability of data

All manuscripts must include a data availability statement. This statement should provide the following information, where applicable:

- Accession codes, unique identifiers, or web links for publicly available datasets
- A list of figures that have associated raw data
- A description of any restrictions on data availability

The raw data for Figs. 1-8 are provided in an Excel file as Source Data.

Field-specific reporting

Please select the one below that is the best fit for your research. If you are not sure, read the appropriate sections before making your selection.

- [x] Life sciences
- [ ] Behavioural & social sciences
- [ ] Ecological, evolutionary & environmental sciences

For a reference copy of the document with all sections, see nature.com/documents/nr-reporting-summary-flat.pdf

Life sciences study design

All studies must disclose on these points even when the disclosure is negative.

Sample size	No sample size calculation was performed. Mouse behavior data for each condition were sampled from 7-8 mice. Slice physiology data for each condition were sampled from 7-8 recorded cells of 3 mice. Immunohistochemical data for each condition were sampled from tissue sections of 3-5 mice. The sample sizes are similar to those reported in previous studies (PMID: 29581428, PMID: 28683263, PMID: 26113723)
Data exclusions	No data were not excluded from the analysis.
Replication	Data of each experiment were acquired and analyzed by two different experimenters blind to the group allocation of the samples. For example, experimenter #1 acquired data set #1 and experimenter #2 acquired data set #2. Then experimenter #1 analyzed data set #2, whereas experimenter #2 analyzed data set #1, totally without knowing which is control or test group. And their results were compared to make sure the results were reproducible. The experiments were independently replicated for 3-8 times, depending on the type of experiments.
Randomization	Randomization was not required because this study did not allocate different mouse experimental groups.
Blinding	All investigators were blinded to group allocation during data collection and analysis.

Reporting for specific materials, systems and methods

We require information from authors about some types of materials, experimental systems and methods used in many studies. Here, indicate whether each material, system or method listed is relevant to your study. If you are not sure if a list item applies to your research, read the appropriate section before selecting a response.

Materials & experimental systems

n/a	Involved in the study	
[ ]	[x]	Antibodies
[x]	[ ]	Eukaryotic cell lines
[x]	[ ]	Palaeontology and archaeology
[ ]	[x]	Animals and other organisms
[x]	[ ]	Human research participants
[x]	[ ]	Clinical data
[x]	[ ]	Dual use research of concern

Methods

n/a	Involved in the study	
[x]	[ ]	ChIP-seq
[x]	[ ]	Flow cytometry
[x]	[ ]	MRI-based neuroimaging

Antibodies

Antibodies used	Rabbit Polyclonal Anti-EGFP: Abcam, CAT# ab290; Lot# GR3196305-l; dilution 1:2000<br>Rabbit Polyclonal Anti-mCherry: Abcam, CAT# ab167453; Lot# GR587698; dilution 1:2000<br>Rabbit Polyclonal Anti-Glutamate: Sigma, CAT# G6642; Lot# 116H4815; dilution 1:500<br>Rabbit Polyclonal Anti-GABA: Sigma, CAT# A2052; Lot# 238K2568; dilution 1:500<br>Rabbit Polyclonal Anti-TH: Sigma, CAT#AB152; Lot# 457842; dilution 1:500<br>Goat anti-Rabbit Alexa Fluor 488 (A11034)<br>Goat Anti Rabbit Alexa Fluor 546 (A11010)

	Goat anti-mouse Alexa Fluor 488 (A11001)<br>Goat Anti Rabbit Alexa Fluor 546 (A11030)
Validation	EGFP: Website states the rabbit polyclonal anti-GFP antibody (ab290) has been tested in flow cytometry, IHC, ICC, IP, and WB and is suitable for ChIP. Reference: PubMed:30510196<br>mCherry: Website states the rabbit polyclonal anti-mCherry antibody is tested for IHC and WB. Reference: PubMed: 29581428<br>Glutamate: Website states the antiserum is evaluated for activity and specificity by dot-blot immunoassay. The antiserum recognizes L-glutamic acid immobilized on an affinity membrane. No cross-reaction is observed with L-aspartic acid, L-glutamine, L-asparagine, and L-alanine. Reference: PubMed 29581428<br>GABA: Website states expression of GABA in neucortical cells harvested from the brains of E19 day old rat embyros was detected by immunofluorescence using rabbit anti-GABA antibody. Reference: PubMed 29581428<br>TH: Website states Anti-Tyrosine Hydroxylase Antibody detects level of TH and has been published and validated for use in ELISA, IF, IH, IH(P), IP and WB. Reference: PubMed 29581428

Animals and other organisms

Policy information about studies involving animals; ARRIVE guidelines recommended for reporting animal research

Laboratory animals	C57BL/6 male mice (3-5 month old) were used in this study: WT mice, GAD2-IRES-Cre mice, vGlut2-IRES-Cre mice, Ai14 mice, and DAT-IRES-Cre mice mice. Mice were maintained on a circadian 12-h light/12-h dark cycle with food and water available ad libitum. The ambient temperature was between 20 and 22 centigrade. The humidity level was between 50%-60%. Mice were housed in groups (3–5 animals per cage) before they were separated 3 days prior to virus injection.
Wild animals	No wild animals were used in this study.
Field-collected samples	No field-collected samples were used in this study.
Ethics oversight	All experimental procedures were conducted following protocols approved by the Administrative Panel on Laboratory Animal Care at the National Institute of Biological Sciences, Beijing (NIBS).

Note that full information on the approval of the study protocol must also be provided in the manuscript.
